# Trigeminal sensitisation by subdural bleeding may mediate brain swelling in acute traumatic brain injury

**DOI:** 10.1186/1129-2377-14-S1-P154

**Published:** 2013-02-21

**Authors:** J Mack, W Squier

**Affiliations:** 1 Penn State Hershey Medical Center, USA; 2 John Radcliffe Hospital , Oxford, UK

## Introduction

Understanding of phenomena such as neurogenic inflammation (NI) and sensitisation of the trigeminovascular system is grounded in migraine research, but may have application in traumatic brain injury (TBI).

## Objectives

Catastrophic brain swelling and poor prognosis seen in TBI associated with small volume subdural bleeding (SDH) is currently unexplained; the mass of SDH is insufficient to explain the swelling. Instead we propose that traumatic dural bleeding and inflammation sensitise the trigeminal system, as demonstrated in animal models, and may mediate brain swelling in TBI.

## Methods

Human autopsy dura was studied by immunocytochemistry for mast cells and for the trigeminal neuropeptides, SP and CGRP.

## Results

Dural bleeding is associated with increased mast cell numbers and altered expression of SP and CGRP. SP expression in dural nerves also varies with age and gender.

## Conclusion

SDH is associated with increased numbers of mast cells, which can cause prolonged excitation in trigeminal meningeal nociceptors. SDH also leads to altered SP and CGRP expression in the dural nerves, indicating trigeminal activation. This has been shown to cause vasodilatation and increased blood flow in the ipsilateral hemisphere, sparing basal ganglia and hindbrain and closely replicating the specific distribution of brain swelling described beneath thin film SDH, particularly in young people. SP variation with age and gender corresponds to patterns of dural pathology; the high female rate of migraine after puberty and the high rate of dural effusions and sinus thrombosis in male infants. Further, vascular leakiness due to trigeminal excitation may explain post-traumatic dural effusions. The role of the trigeminovascular system in the cascade of brain injury following trauma has not been explored and we believe there is an urgent need for the wealth of information derived from migraine research to be applied to the study of brain trauma. This may point to new treatments that could reduce the morbidity and mortality in acute head trauma associated with SDH.
